# Clonal Hematopoiesis at the Crossroads of Inflammatory Bowel Diseases and Hematological Malignancies: A Biological Link?

**DOI:** 10.3389/fonc.2022.873896

**Published:** 2022-04-12

**Authors:** Cosimo Cumbo, Francesco Tarantini, Antonella Zagaria, Luisa Anelli, Crescenzio Francesco Minervini, Nicoletta Coccaro, Giuseppina Tota, Luciana Impera, Elisa Parciante, Maria Rosa Conserva, Immacolata Redavid, Paola Carluccio, Mario Delia, Annamaria Giordano, Maria Chiara Longo, Tommasina Perrone, Antonella Russo Rossi, Giorgina Specchia, Pellegrino Musto, Francesco Albano

**Affiliations:** ^1^ Department of Emergency and Organ Transplantation (D.E.T.O.), Hematology and Stem Cell Transplantation Unit, University of Bari “Aldo Moro”, Bari, Italy; ^2^ School of Medicine, University of Bari “Aldo Moro”, Bari, Italy

**Keywords:** clonal hematopoiesis, inflammatory bowel diseases, hematological malignancies, *DNMT3A*, *ASXL1*, *JAK2*

## Abstract

Inflammatory bowel diseases (IBDs) are a group of chronic conditions of the gastrointestinal tract in which nationwide studies have revealed a higher risk of hematological malignancies (HMs). Clonal hematopoiesis (CH) is a premalignant condition defined by the presence of an acquired somatic mutation characterized by a variant allele frequency (VAF) of ≥2%, in a gene frequently associated with HMs. A growing body of evidence suggests a correlation between inflammation and CH; its occurrence in the context of IBD has been previously demonstrated. With the aim to assess CH possible co-occurrence in patients with an IBD associated with HMs, we performed a targeted next-generation sequencing analysis in a cohort of thirteen patients who were referred to our center with IBD associated with HMs. Eleven (85%) patients showed one or more mutations in CH-associated genes; *DNMT3A* was the most frequently mutated gene, followed by *ASXL1* and *JAK2.* These results may suggest that the mechanisms at the basis of the inflammatory environment could potentially select for the growth of hematopoietic clones harboring specific mutations. In this context, CH emergence may be boosted by the proinflammatory IBD environment, thus acting as a biological link between IBD and the HM onset. If these data are confirmed, IBD patients screened and positive for CH should undergo a hematologic follow-up to assess the risk of developing HM. Future study will clarify the relationship between these conditions.

## Introduction

The term inflammatory bowel diseases (IBDs) define a group of chronic conditions of the gastrointestinal tract. Crohn’s disease (CD) and ulcerative colitis (UC) are the most common types. These two conditions differ slightly, both histologically and clinically. IBDs are thought to arise due to host genetics, environmental factors, and immune system function, ultimately resulting in chronic inflammation and consequent clinical symptoms (1). Nationwide studies have revealed a higher risk of hematological malignancies (HMs) in these patients (2–4). In particular, IBDs are associated with the onset of lymphoproliferative disorders. The hypothetical causal link relies on the iatrogenic effect of novel immunosuppressive drugs (such as thiopurines, methotrexate, and biologic agents) adopted to treat IBDs. Notwithstanding, no clear evidence related to specific drugs has emerged (5).

Clonal hematopoiesis (CH) is a premalignant condition defined by the presence of an acquired somatic mutation characterized by a variant allele frequency (VAF) of ≥2%, in a gene frequently associated with HMs, without fulfillment of other diagnostic criteria for a hematologic neoplasm diagnosis (6). CH incidence is age-related, showing a prevalence of about 10% in persons aged > 70 years, while it is less frequent in young and young adults. A growing body of evidence suggests a correlation between inflammation and CH. On the one hand, an inflammatory state may favor the selection and consequent emergence of the mutated hematopoietic clone (7), thus acting as a stress factor on hemopoiesis. On the other hand, the mutated clone itself may increase the expression of inflammatory genes in innate immune cells (8).

IBD patients have an underlying pro-inflammatory state, whose potential effects could intersect the roads of CH, as already demonstrated in atherosclerosis and autoimmune disorders, such that these diseases could boost the emergence of CH (9, 10). Recently, experimental data are unveiling ever more profound interactions between a pro-inflammatory milieu and mutated hematopoietic stem cells (HSCs). These latter show a sort of adaptive capacity, not only conferring resistance to inflammation but ultimately enhancing their ability to outgrow normal HSCs (11).

All this premised, a reflection is needed to investigate the link between IBDs and HMs. Conversely, an iatrogenic trigger (i.e., drugs) could be enough to cause a neoplastic evolution. Moreover, the interplay between the hematopoietic system and inflammation could be more than a supporting cast.

## Method

From February 2011 to May 2021, 13 cases (median age 58 years, range 47 – 70) were referred to our center with IBD associated with HMs; their main clinical data are reported in **Table 1**. In all cases, the IBD diagnosis was confirmed by histological analysis. Before the HM onset, they were all treated with mesalazine except case #5. The study was approved by the local ethics committee “Azienda Ospedaliero Universitaria Policlinico di Bari”. Written, informed consent was obtained from all patients before enrolment in accordance with the Declaration of Helsinki. Their records/information were anonymized and de-identified before analysis.

**Table 1:** Annotation of variants identified in IBD cohort.

Case	Sex/Age	IBD	HM	Time from IBD to HM (months)	Gene	Locus	Protein	Location	Function	VAF(%)	MAF	COSMICv95	dbSNP
#1	M/65	UC	CML	19*	*GATA2*	chr3:128204933G>C	p.Leu170Val	exon 3	missense	44.12	0	6939682	//
#2	M/53	UC	CLL	75*	*EZH2*	chr7:148524337C>T	p.Arg216Gln	exon 7	missense	38.46	0.000007	6657582	rs747028969
					*JAK2*	chr9:5072561G>A	p.Gly571Ser	exon 13	missense	48.82	0.000461	29107	rs139504737
#3	M/59	UC	AML	26	*DNMT3A*	chr2:25467449C>A	p.Gly543Cys	exon 14	missense	53.05	0	87002	rs752222356
#4	M/65	UC	CLL	74	*DNMT3A*	chr2:25462023C>T	p.Trp795Ter	exon 20	nonsense	3.07	0	//	rs756566100
#5	M/47	CD	MDS	4	*ASXL1*	chr20:31022835A>T	p.Arg774Ter	exon 12	nonsense	29.36	0	4385101	rs764604832
					*ETV6*	chr12:12038879A>G	p.Tyr391Cys	exon 7	missense	13.36	0	5748387	//
						chr12:12037412T>A	p.Leu348Ter	exon 6	nonsense	6.75	0	//	//
#6	F/70	UC	DLBCL	48	*DNMT3A*	chr2:25469028C>T	p.?	exon 11	splice site	3.82	0	5945645	//
#7	F/50	UC	T-NHL	127	*ASXL1*	chr20:31026380G>A	p.?	exon 12	3’-UTR	51.93	0	60125843	rs112187626
					*DNMT3A*	chr2:25505541A>G	p.Ser73Pro	exon 4	missense	2.59	0	//	rs758401672
#8	F/49	CD	CML	156	*GATA2*	chr3:128199830G>A	p.?	exon 6	3’-UTR	49.57	0.000114	//	rs374495352
					*KIT*	chr4:55593431G>A	p.Val530Ile	exon 10	missense	48.05	0.000594	1155	rs72550822
#10	F/66	UC	CLL	10*	*SF3B1*	chr2:198267492T>A	p.Glu622Val	exon 14	missense	9.86	0	1159839	//
					*JAK2*	chr9:5126343G>A	p.Arg1063His	exon 24	missense	48.50	0.00470	6495318	rs41316003
					*ASXL1*	chr20:31023702C>T	p.Gln1063Ter	exon 12	nonsense	10.45	0	159235	rs1311033207
					*ASXL1*	chr20:31024704G>A	p.Gly1397Ser	exon 12	missense	46.32	0.00188	133033	rs146464648
#11	M/50	UC	CLL	71*	*JAK2*	chr9:5123108A>G	p.Lys1055Arg	exon 23	missense	48.72	0.0000041	4384410	rs1349849518
#12	M/58	CD	NHL	17*	*TET2*	chr4:106157698T>C	p.Tyr867His	exon 3	missense	51.90	0.00713	327337	rs144386291
					*TET2*	chr4:106196834C>T	p.Pro1723Ser	exon 11	missense	51.48	0.00697	1235472	rs146348065

*Diagnosis of IBD was made after that of hematological disease; symptoms of IBD were present before the HM diagnosis.

Case#9 and #13, reporting no gene variants, were not included in this table.

IBD, inflammatory bowel disease; UC, ulcerative colitis; CD, Crohn’s disease; HM, hematological malignancy; CML, chronic myeloid leukemi;, CLL, chronic lymphocytic leukemia; AML, acute myeloid leukemia; MDS, myelodysplastic syndrome; DLBCL, diffuse large B-cell lymphoma; T-NHL, T-cell non-Hodgkin’s lymphoma; NHL, non-Hodgkin’s lymphoma; VAF, variant allele frequency; MAF, minor allele frequency (gnomAD v2.1.1).

Next-generation sequencing (NGS) analysis with an AmpliSeq customized panel (Thermo Fisher Scientific), encompassing 26 target genes that are frequently mutated in myeloid malignancies, was performed on genomic DNA extracted from bone marrow (BM) (cases #3 - #13) or peripheral blood (PB) (cases #1, #2) samples, as previously described (12). Quality control reads alignment to the human genome (hg19) and variant calling (using the somatic workflow for single samples and the default parameters) were performed using Torrent Suite Software v5.16 (Thermo Fisher Scientific). Variants were annotated using Ion Reporter Software v5.16 (Thermo Fisher Scientific). Variants located in intronic regions (not in splice sites) or synonymous or present with ≥1% global minor allele frequency (MAF) in the healthy population, according to the Genome aggregation database (gnomAD) v2.1.1, were filtered out. Only variants affecting the CH-associated genes (13), with ≥2% VAF (6), and with a depth of coverage >500x were considered. Selected variants were investigated for a potential pathogenic role using the Catalogue of Somatic Mutations in Cancer (COSMIC v95) and dbSNP databases.

## Results

**Figure 1:**
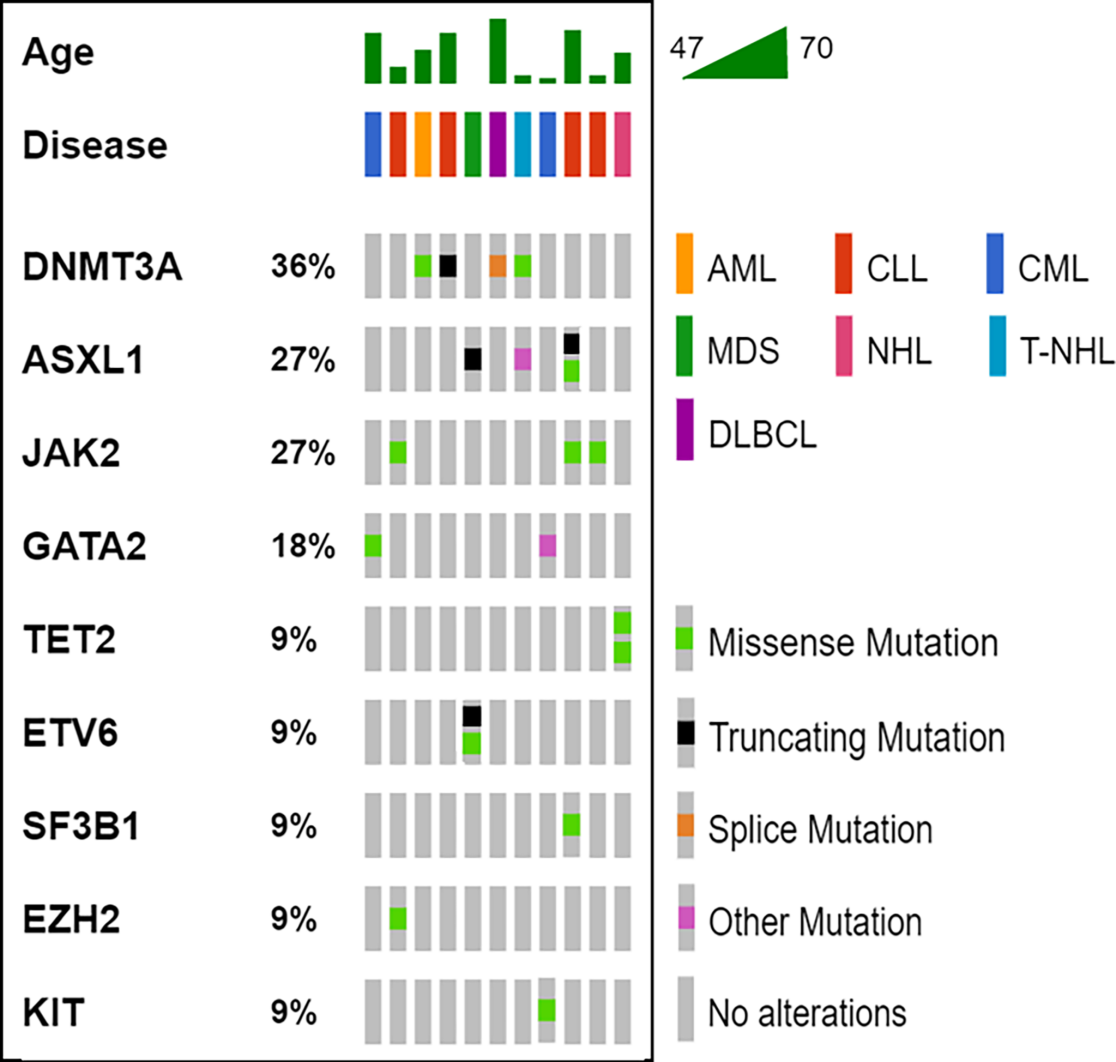
Oncoprinter visualization of all variants identified. For all cases (columns), the age, the hematological disease and the variants identified are reported. The percentage value associated to each gene, indicates its variants occurrence in the cohort analyzed. AML, acute myeloid leukemia; CLL, chronic lymphocytic leukemia; CML, chronic myeloid leukemia; DLBCL, diffuse large B-cell lymphoma; MDS, myelodysplastic syndrome; NH, non-Hodgkin’s lymphoma; T-NHL, T-cell non-Hodgkin’s lymphoma.

The median latency time between IBD and HM was 71 months (range 4 – 312 months). Overall, 20 variants affecting 9 genes (*DNMT3A*, *ASXL1*, *JAK2*, *GATA2*, *TET2*, *ETV6*, *SF3B1*, *EZH2*, *KIT*) were detected in our cohort (Table and **Figure 1**); 11/13 (85%) patients showed one or more mutations, with a VAF ranging from 2.6% to 53.0%. Moreover, cases #6 and #7 showed variants of CH-associated genes but in the absence of a BM lymphoma localization. Five variants (25.0%) showed a VAF<10%, whereas, in the 15 others identified (75.0%), the clone was dominant. In accordance with previously published data in the IBD context, *DNMT3A* was the most frequently mutated gene (4/20, 20.0%) (13, 14), with *ASXL1* (4/20, 20.0%), followed by *JAK2* (3/20, 15.0%). All mutations are single nucleotide variants (SNV) with different functions: missense variants (13/20, 65.0%), nonsense variants (4/20, 20.0%), and unknown variants affecting a splice site or an untranslated region (3/20, 15.0%). Five cases (45.5%) carried just one variant, whereas the other six (54.5%) showed two or more mutations.

## Discussion

**Figure 2:**
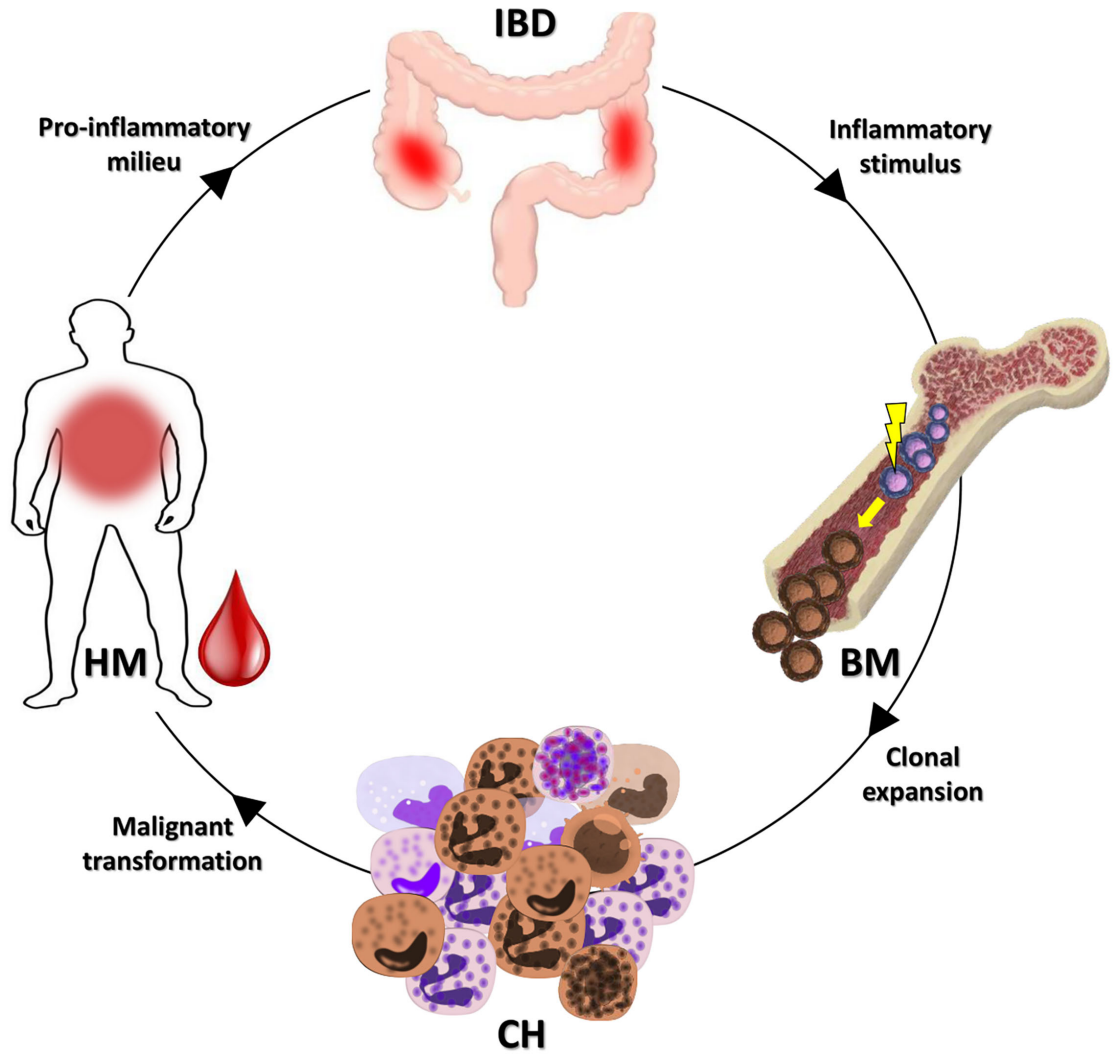
Possible link between IBDs and HMs. As demonstrated, an IBD may promote the CH onset (14). The clone selected could evolve in a subsequent HM or favor the insurgence of an HM. In both cases, the inflammatory milieu of the HM may, in turn, feed the IBD. IBD, inflammatory bowel disease; BM, bone marrow; CH, clonal hematopoiesis; HM, hematological malignancy.

Individuals with CH have a higher incidence of HM, coronary heart disease, ischemic stroke, and heart failure (15). Recent works demonstrated that chronic infection, UC and atherosclerosis, can drive the gene loss of function associated with CH (14, 16, 17). In particular, chronic infection and UC may promote the selection of the *DNMT3A* gene mutation associated with CH by the IFNγ signaling induced in the course of these disorders (14, 16). It is worthy of note that four (36.4%) cases in our series bore *DNMT3A* gene mutations. According to data previously shown in UC patients (14), this fact may be considered to demonstrate that the mechanisms at the basis of the inflammatory environment potentially select for the growth of CH specific mutations. Thus, CH may act as a possible biological link between IBD and the HMs onset (**Figure 2**). In fact, CH may be fed by the IBD inflammatory stimuli, and in turn, feed the IBD inflammation. This context can also explain the low median age of our patient series compared to that expected in healthy individuals with CH.

Our main study limitation is that it was not possible to demonstrate that the CH-associated genes variant was present before the onset of the HMs, since no genomic data were available at that time. Notwithstanding, the low median age of our patients, the prevalence of lymphoproliferative disorders (not due to mesalazine administration), and the incidence of typical CH-associated mutations in our cohort seem to suggest a biological link between these conditions.

Interestingly, in our observation 4 patients (#4, #5, #6, and #7) showed a very low VAF of the CH-associated genes. This circumstance could suggest that there may be a distinct role between CH promoted and selected by inflammation and the genomic alterations that drive tumorigenesis and control the HM pathogenesis. Particularly, the low VAF of *DNMT3A* gene in cases #6 and #7, both patients without evidence of BM lymphoma involvement, is strongly suggestive of CH promoted by IBD. It’s noteworthy that *DNMT3A* mediated CH should not be seen as an inactive player in the immune system. In fact, it has been shown to alter T cells’ homeostasis and be associated with enhanced T cell activity in recipients of allografts harboring a *DNMT3A* mutation (18, 19).

Furthermore, cases #1, #2, #8, #10, #11 showed CH-associated gene variants usually rare in chronic myeloid and lymphoid leukemia. It’s noteworthy that we didn’t include two cases of *JAK2* V617F myeloproliferative neoplasms in our analysis, preceded by IBD. Both patients were aged <70 and had a multiannual story of IBD at the time of HM onset. In this context, we should consider that, on the one hand, *JAK2* is undoubtedly “driving” the disease; on the other hand, it accounts for 3% of CH in the general population (20). Therefore, even in these circumstances, the interplay between IBD, CH and HM cannot be *a priori* excluded.

In conclusion, our report suggests that CH may be seen as a biological link at the crossroads of IBDs and HMs, whose role is yet to be fully elucidated. Nonetheless, if these data are confirmed, IBD patients screened and positive for CH should undergo hematologic follow-up to assess the risk of developing HM. Further studies are warranted to characterize the biological relationship between IBDs, CH, and HMs.

## Data Availability Statement

The datasets presented in this study can be found in online repositories. The names of the repository/repositories and accession number(s) can be found below: https://www.ncbi.nlm.nih.gov/bioproject/PRJNA719027.

## Ethics Statement

The studies involving human participants were reviewed and approved by Local ethics committee “Azienda Ospedaliero Universitaria Policlinico di Bari”. The patients/participants provided their written informed consent to participate in this study.

## Author Contributions

CC, FT, and FA conceived and designed the study and wrote the manuscript. CC and AZ performed the main experiments. LA, CM, NC, GT, LI, EP, MC, and IR performed diagnostic molecular analysis. PC, MD, AG, MCL, TP, and AR provided clinical data. GS, PM, and FA reviewed and edited the manuscript. FA supervised the manuscript preparation. All authors contributed to the article and approved the submitted version.

## Funding

This work was supported by “Associazione Italiana contro le Leucemie (AIL)-BARI” and by the association for non-Hodgkin lymphoma research “Il sorriso di Antonio”, Corato – Italy.

## Conflict of Interest

The authors declare that the research was conducted in the absence of any commercial or financial relationships that could be construed as a potential conflict of interest.

## Publisher’s Note

All claims expressed in this article are solely those of the authors and do not necessarily represent those of their affiliated organizations, or those of the publisher, the editors and the reviewers. Any product that may be evaluated in this article, or claim that may be made by its manufacturer, is not guaranteed or endorsed by the publisher.
